# How Is Virtuous Personality Trait Related to Online Deviant Behavior among Adolescent College Students in the Internet Environment? A Moderated Moderated-Mediation Analysis

**DOI:** 10.3390/ijerph19159528

**Published:** 2022-08-03

**Authors:** Heyun Zhang, Huanhuan Zhao

**Affiliations:** Department of Psychology, Shanghai Normal University, Shanghai 200234, China; zhangheyun@shnu.edu.cn

**Keywords:** online deviant behavior, virtuous personality trait, moral disengagement, perspective taking, moderated moderated-mediation model

## Abstract

Online deviant behavior is a series of maladaptive behaviors that may have adverse effects on the physical and mental health of others and adolescents in the Internet environment. Previous studies have paid more attention to the risk factors of adolescent online deviant behavior. However, its protective factors and psychological mechanisms remain unclear. Thus, this study explored the protective effect of virtuous personality trait on adolescents’ online deviant behavior and its psychological mechanism. A total of 851 Chinese college students anonymously completed a series of questionnaires about virtuous personality trait, moral disengagement, perspective taking, and online deviant behavior. The findings showed the following: (1) Virtuous personality trait was negatively correlated with online deviant behavior. (2) Moral disengagement mediated the relationship between virtuous personality trait and online deviant behavior. (3) Perspective taking moderated the first half stage of the mediation model in which college students’ virtuous personality trait influences online deviant behavior via moral disengagement. (4) A moderated moderated-mediation analysis found that gender moderated the moderating effect of perspective taking on the relationship between virtuous personality trait and moral disengagement. This study is helpful to demonstrate the protective effect and psychological mechanism of virtuous personality trait on online deviant behavior. Some theoretical and practical significance and limitations were also analyzed and discussed.

## 1. Introduction

Environment can affect people’s psychology and behavior. Compared with other human environments, the Internet is a new environment, which has become an important factor affecting people’s psychology and behavior. With the popularization of the Internet, Internet users are increasing, and the Internet has had an extensive, profound, and lasting impact on human society. As of December 2021, the number of China’s Internet users had reached 1032 million. Among them, children and adolescents have become the main group of Internet users, accounting for approximately one-third of the total [1]. On the one hand, for children and adolescents, cyberspace is full of learning resources and opportunities to promote their own development. On the other hand, cyberspace is also full of all kinds of risks and temptations, causing their own deviant behaviors [2]. In recent years, adolescents’ online deviant behaviors, such as cyber flaming behavior, cyberbullying behavior, cyber obscenity/pornography, and deception on the Internet, have increasingly attracted the attention of researchers [3,4,5,6]. College students are in a period of late adolescence when their outlook on the world, life, and values have not yet been established. Their thoughts and behaviors are easily influenced by other negative phenomena. In the chaotic Internet environment, adolescents are more likely to exhibit some online deviant behaviors [7,8]. Online deviant behavior is considered a series of maladaptive behaviors that will have many adverse effects on adolescents in the Internet environment [4,7,9]. Many studies have shown that online deviant behavior is strongly associated with substance use, academic problems, mental disorders, illegal crimes, and so on [6,10,11,12].

Therefore, it is necessary to investigate the influencing factors and psychological mechanism of online deviant behavior so as to provide effective intervention strategies for reducing online deviant behaviors in adolescent college students.

### 1.1. Virtuous Personality Trait and Online Deviant Behavior

Given the negative impact of online deviant behavior on adolescents’ growth, more and more researchers are paying attention to reducing online deviant behavior in adolescents. The external factors of family and society that influence online deviant behavior have been primarily explored [4,6,13,14], but the internal personality factors of individuals were given less attention. According to problem behavior theory, the individual system and the perceived social environment system are two important systems that affect the development of adolescents’ problem behavior [15]. As an important research theme of the individual system, personality trait is considered to be an important factor affecting online deviant behaviors [6,8]. Previous research has revealed that some “negative” personality variables (i.e., dark triad traits, impulsivity, and neuroticism) are considered as risk factors that can increase online deviant behaviors [6,16,17]. In addition, according to the theory of positive youth development [18,19,20], individuals have inherent strengths and potentials (e.g., character, caring/compassion) that contribute to their healthy development, which can help them reduce problem behaviors. Virtuous personality trait is one kind of moral character traits [21], also called the kindness trait, which embodies the overall characteristics of “good people”, including integrity, altruism, tolerance, faithfulness, and care for others [21,22,23]. This “positive” personality trait may play a protective role in reducing adolescents’ online deviant behaviors. Nevertheless, so far, there is no direct evidence to reveal the relationship between adolescents’ virtuous personality trait and online deviant behavior. However, previous studies have pointed out that people with high levels of virtuous personality trait tend to exhibit high levels of honesty, agreeableness, conscientiousness, and empathy for others [21,23]. Additionally, some studies also indicated that these types of personality traits (e.g., agreeableness, conscientiousness) can significantly negatively predict online deviant behaviors [8,17]. In addition, previous studies have also pointed out that people with a low score on virtuous personality trait are dishonest, hypocritical, deceptive, and unscrupulous [21,23], which may mean that individuals with low levels of virtuous personality trait may have more online deviant behaviors.

From what has been discussed above, we inferred that adolescent college students’ virtuous personality trait could be considered as a protective factor that can decrease online deviant behavior.

### 1.2. Moral Disengagement as a Mediator

Above, we have discussed the possible relationship between virtuous personality trait and online deviant behavior. However, the potential influencing mechanism of virtuous personality trait on adolescent college students’ online deviant behavior is unclear. Through a review of previous literature, we found that moral disengagement may be an important psychological mechanism [24,25,26]. Moral disengagement, proposed by Bandura [27], refers to a series of psychological strategies (e.g., dehumanization, diffusion of responsibility, moral justification, etc.) used by individuals to dissociate their internal moral standards from their actions, facilitating individual engagement in unethical behaviors without feeling self-sanctioning emotions (e.g., guilt, remorse, and shame) [27,28]. Based on Bandura’s theory of moral disengagement, Yang et al. [25] found that the moral disengagement of college students can significantly positively predict online deviant behavior. Some meta-analysis studies have also confirmed that moral disengagement can predict adolescents’ online deviant behavior significantly positively [24]. Thus, moral disengagement as a risk factor for adolescents’ online deviant behaviors is natural.

As a moral character, the virtuous personality trait is closely related to high integrity, high moral standards, high agreeableness, high conscientiousness, high altruism, and so on [5,21,23]. People with virtuous personality trait tend to have more integrity and high moral standards, which may lead them to exhibit a lower level of moral disengagement in the Internet environment. Although no research has directly revealed the relationship between virtuous personality trait and moral disengagement, previous studies have identified that virtuous personality-related traits (e.g., agreeableness and conscientiousness) can significantly negatively predict individuals’ moral disengagement [29,30]. Thus, we speculate that virtuous personality trait can negatively predict adolescents’ moral disengagement in theory.

On the basis of the above discussion, virtuous personality trait in adolescents can be negatively associated with moral disengagement, which is positively correlated with their online deviant behavior. Accordingly, we propose that adolescent college students’ moral disengagement may mediate the relationship between virtuous personality trait and online deviant behavior.

### 1.3. Perspective Taking as a Moderator

Moral disengagement may play a mediating role in the relationship between adolescents’ virtuous personality trait and online deviant behavior. This study is of great significance to explore the protective effect and psychological mechanism of adolescents’ virtuous personality trait on their online deviant behavior. Nevertheless, when this protective effect and psychological mechanism is stronger or weaker is unclear. The current study wanted to test a hypothesis that the association between virtuous personality trait and moral disengagement would be moderated by adolescent perspective taking. Previous studies have suggested that perspective taking is the cognitive component of empathy or the ability of an individual to put himself in others’ shoes and infer others’ internal psychological activities [31,32]. Some studies have shown that perspective taking can make adolescents consider things from the perspective of others and show less moral disengagement in the Internet world [33,34,35]. In addition, previous studies have also found that empathy contributes to moral judgments [36], which means that empathy can make individuals more likely to behave consistently with their moral standards, thus inhibiting moral disengagement. From this evidence, it can be found that the individual’s ability of perspective taking can make the individual exhibit more kindness tendency, and then show less moral disengagement. Previous empirical studies have demonstrated that perspective taking can moderate the relationship between some personality traits (e.g., dark triad personality traits, callous-unemotional traits, etc.) and adolescents’ moral disengagement [34,37]. This evidence from previous studies suggests that perspective taking may enhance the negative predictive effect of virtuous personality trait on adolescent college students’ moral disengagement.

Thus, we propose that adolescent college students’ perspective taking may play a moderating role in the relationship between virtuous personality trait and moral disengagement, and the relationship is stronger when perspective taking is higher.

### 1.4. Gender as a Moderator

Previous studies have indicated that moral delinquents tend to make self-serving decisions, and exhibit fewer altruistic, friendly behaviors [27,29,38], which can be detrimental to interpersonal relationships. Evolutionary theories imply that, to raise children successfully, women tend to be more cautious, kind, and emotionally involved [39]. Numerous studies have consistently found that women score higher than men on certain personality traits related to kindness (i.e., agreeableness) [40,41,42]. Miller’s relational theory has pointed out that women care more about interpersonal relationships [33,43]. Consequently, for women, they have a relatively higher level of virtuous personality trait and a lower level of moral disengagement, which means that women’s virtuous personality trait may have a stronger negative predictive effect on moral disengagement. Therefore, gender may moderate the relationship between adolescents’ virtuous personality trait and moral disengagement. Specifically, in order to construct a good interpersonal relationship, women with high virtuous personality trait may show less moral disengagement than men.

Furthermore, as mentioned above, adolescents’ perspective taking may enhance the negative predictive effect of virtuous personality trait on moral disengagement. Previous studies have also demonstrated gender differences in virtuous personality trait, perspective taking, and moral disengagement. To be specific, compared with men, women have higher virtuous personality traits and perspective taking, and lower moral disengagement [40,41,44]. According to Miller’s relational theory, women have a stronger desire to develop and maintain interpersonal relationships than men [33,43]. Thus, adolescents’ gender may also moderate the moderating effect of perspective taking on the link between virtuous personality trait and moral disengagement. That is, women with high levels of perspective taking may exhibit less moral disengagement than men, even though they have low virtuous personality trait. However, men with low levels of perspective taking may show more moral disengagement and have lower virtuous personality trait [40,41]. Therefore, the relationship between adolescents’ virtuous personality trait and moral disengagement would become much weaker for men with low levels of perspective taking.

To sum up, we propose that adolescent college students’ gender may moderate the moderating effect of perspective taking on the relationship between virtuous personality trait and moral disengagement.

### 1.5. The Present Study

**Figure 1:**
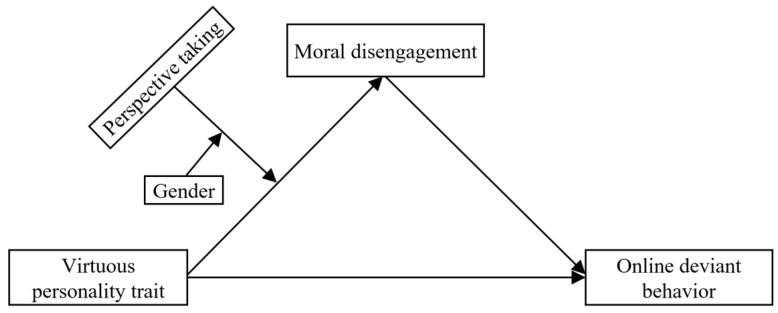
The proposed moderated moderated-mediation model.

Previous studies have paid more attention to the risk factors of adolescent online deviant behavior. However, its protective factors and psychological mechanisms remain unclear. Thus, based on the theory of positive youth development, this study selected college students as research objects, tried to explore the protective effect of virtuous personality trait on adolescents’ online deviant behavior, and revealed its psychological mechanism by constructing a moderated moderated-mediation model (see Figure 1). The main specific research questions are as follows: First, the current study tested whether adolescents’ virtuous personality trait can be used as a protective factor to reduce their online deviant behavior in the Internet environment. Second, the current study tested whether adolescents’ moral disengagement will mediate the relationship between virtuous personality trait and online deviant behavior. Third, the current study tested whether perspective taking will moderate the link between adolescents’ virtuous personality trait and moral disengagement. Last, the current study tested whether gender moderates the moderating effect of perspective taking on the association between adolescents’ virtuous personality trait and moral disengagement.

Accordingly, this study tested the following hypotheses:

**Hypothesis** **1.** **(H1).** *Adolescent college students’ virtuous personality trait can negatively predict their online deviant behavior.*

**Hypothesis** **2.** **(H2).** *Moral disengagement plays a mediating role between virtuous personality trait and online deviant behavior.*

**Hypothesis** **3.** **(H3).** *Perspective taking plays a moderating role in the relationship between virtuous personality trait and moral disengagement.*

**Hypothesis** **4.** **(H4).** *Adolescent college students’ gender can moderate the moderating effect of perspective taking on the relationship between virtuous personality trait and moral disengagement. In other words, the moderated moderated-mediation model constructed in this study is valid.*

## 2. Method

### 2.1. Participants

A total of 879 college students were recruited from public classes (e.g., psychology and life, psychology and education, and psychological health course) in several Chinese universities by convenience sampling method. The data collection work was carried out by the teachers or counselors of the corresponding public psychology courses. From October to December 2020, the link of the questionnaire was sent to the students through the Questionnaire Star (a popular Chinese website for the online survey), and the students completed the questionnaire after informed consent. After preliminary screening and analysis of the data, twenty-eight of the subjects were excluded because they answered regularly or failed to pass the attitude test questions. The final sample comprised of 851 Chinese college students (267 men, 584 women) aged from 18 to 24 years old (*M* = 20.59, *SD* = 1.08). Of all the participants, 5.30% were freshmen (*N* = 45), 14.60% sophomores (*N* = 124), 51.90% juniors (*N* = 442), and 28.20% seniors (*N* = 240).

### 2.2. Measures

#### 2.2.1. Virtuous Personality Trait

The Chinese virtuous personality questionnaire [21] was used to assess adolescents’ level of virtuous personality trait. The questionnaire is a 33-item self-report questionnaire, which comprises four dimensions: integrity and kindness (15 items), altruism and dedication (9 items), tolerance and geniality (6 items), and affection and faithfulness (5 items). Participants responded to the items through a five-point Likert scale (1 = very much unlike me, 5 = very much like me). The higher the aggregated score was, the higher the level of adolescents’ virtuous personality trait. In current study, the Cronbach’s alpha of this questionnaire was 0.94.

#### 2.2.2. Moral Disengagement

The Chinese version of the moral disengagement scale [45] was used to assess the level of adolescents’ moral disengagement, which was adapted from the moral disengagement scale compiled by Bandura et al. [46]. This scale comprises 26 items (e.g., “It is alright to fight to protect your friends.”), which are divided into eight dimensions: moral justification (4 items), euphemistic language (3 items), advantageous comparison (3 items), displacement of responsibility (3 items), diffusion of responsibility (4 items), distorting consequences (3 items), dehumanization (3 items), and attribution of blame (3 items). Participants were asked to indicate the extent to which they agree or disagree with each item on a five-point Likert scale (1 = strongly disagree, 5 = strongly agree). The Cronbach’s alpha of this scale in the current study was 0.85.

#### 2.2.3. Perspective Taking

The current study used the perspective taking subscale of the Interpersonal Reactivity Index [47] to measure the level of adolescent perspective taking. This subscale consists of 5 items (e.g., “When I’m angry with someone, I usually try to think about their position.”). Participants responded to the items through a 5-point Likert scale (1 = not at all like me, 5 = very much like me), and their responses were summed to indicator the level of perspective taking. The Cronbach’s α of this scale was 0.70.

#### 2.2.4. Online Deviant Behavior

Adolescents’ online deviant behavior was assessed by using the Scale for Adolescent Internet Deviance [48]. This self-report questionnaire has 35 items and three dimensions: cyber flaming behavior (20 items; e.g., “When I communicate with others online, I easily conflict with them.”), cyber obscenity/pornography behavior (9 items; e.g., “I have downloaded/watched porn on the Internet.”), and cyber deceptive behavior (6 items; e.g., “I think it’s interesting to cheat people on the Internet.”). Participants replied to each question on a five-point scale ranging from 1 (never) to 5 (always). The higher a participant’s score, the higher their level of online deviant behavior. In current study, the Cronbach’s α of this scale was 0.92.

### 2.3. Procedure

This survey was reviewed and approved by the relevant ethics committee of the author’s university. All participants participated in the study voluntarily and were free to withdraw from the study. All participants initially signed an informed consent form and then completed all self-reported questionnaires independently and anonymously within 20 min. After completing the questionnaire, the participants were thanked.

### 2.4. Statistical Analysis

In this study, SPSS 22.0 software package (IBM, Armonk, NY, USA) and the PROCESS Macro for SPSS [49] were used for data analysis. First, to check for common method bias, this study applied the Harman’s single-factor test with exploratory factor analysis. Second, to perform descriptive analysis and test hypothesis 1, descriptive statistics and correlation analysis were calculated for all variables. Third, to test the mediating effect of moral disengagement in the relationship between virtuous personality trait and online deviant behavior, the Model 4 of the PROCESS macro for SPSS was adopted. Finally, to test the moderated-mediation model and moderated moderated-mediation model, the Model 7 and Model 11 were employed. All the variables were standardized, and the bias-corrected bootstrapping method (resamples = 5000) was used to determine the significance of indirect effects in the models. If the 95% bias-corrected bootstrap confidence interval (CIs) does not contain 0, it means that the indirect effect of the models is significant.

## 3. Results

### 3.1. Common Method Deviation Test

In this study, all data were collected using the self-report method of the subjects, which may lead to common method bias. Therefore, Harman’s single-factor test with an exploratory factor analysis was used in this study [50]. The results showed that there were 24 principal components with eigenvalues of more than 1, and the first factor explained 15.52% of the total variance variation, far less than the critical value of 40%. Therefore, there is no significant common method bias effect in this study.

### 3.2. Descriptive Analyses

The mean values, standard deviations, skewness, kurtosis, and correlation coefficients among the major variables are presented in Table 1. The results showed that the absolute values of Skewness and Kurtosis of all variables were less than 2 and 7 respectively, which means that all variables were normally distributed [51]. Virtuous personality trait was significantly negatively associated with moral disengagement and online deviant behavior, but positively related to perspective taking. Moral disengagement was positively related to online deviant behavior but negatively associated with perspective taking. Therefore, higher scores on virtuous personality trait indicate lower online deviant behavior. Hypothesis 1 is supported.

### 3.3. Testing for the Mediating Role of Moral Disengagement

This study explored the mediating role of moral disengagement between virtuous personality trait and online deviant behavior to reveal how virtuous personality trait influences college students’ online deviant behavior. This mediation model was tested with the macro program PROCESS for SPSS (Model 4; Hayes, 2013). The results are presented in Table 2. After controlling for age and grade, the results revealed that virtuous personality trait was negatively related to moral disengagement (*β* = −0.23, *t* = −7.00, *p* < 0.001), which in turn was positively related to online deviant behavior in adolescents (*β* = 0.41, *t* = 13.24, *p* < 0.001). The negative direct association between virtuous personality trait and college students’ online deviant behavior remained significant (*β* = −0.17, *t* = −5.43, *p* < 0.001). Thus, moral disengagement partially mediated the relationship between virtuous personality trait and college students’ online deviant behavior (indirect effect = −0.10, SE = 0.02, 95% CI = [−0.13, −0.07]). The mediation effect accounted for 36.23% of the total effect of virtuous personality trait and online deviant behavior. Thus, Hypothesis 2 is supported.

### 3.4. Testing for the Moderated-Mediation Model

This study analyzed Hypothesis 3 by examining whether perspective taking would moderate the path between virtuous personality trait and moral disengagement using Model 7 of Hayes’s macro PROCESS for SPSS [49,52]. As shown in Table 3 (see Model 1), the effects of virtuous personality trait on college students’ moral disengagement were significant (*β* = −0.21, *p* < 0.001), and the interaction of virtuous personality trait and perspective taking had a significant predictive effect on moral disengagement (*β* = −0.09, *p* < 0.01). Furthermore, the index shows that the effect of perspective taking is significant in the moderated-mediation relationship (Index = −0.04, SE (Boot) = 0.01, 95% CI = [−0.06, −0.02]). A significant moderation effect was observed by considering perspective taking as a moderator. 

**Figure 2:**
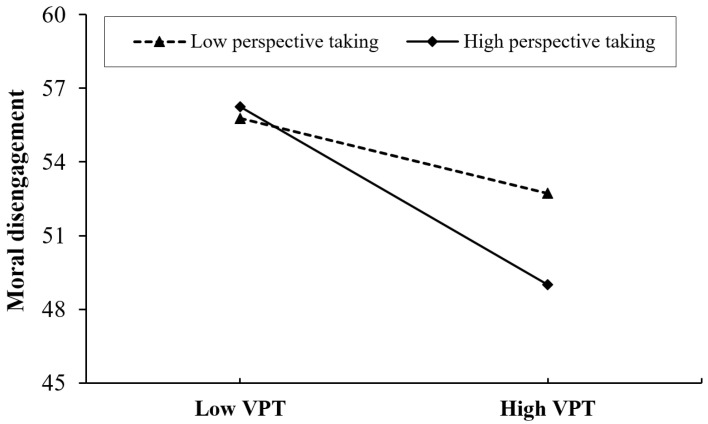
Simple slopes analysis of PT as a moderator in the association between VPT and MD.

This study presented virtuous personality trait on moral disengagement at different levels of perspective taking (M − 1 SD and M + 1 SD) to describe the moderating effect. Simple slope tests (see Figure 2) showed that virtuous personality trait significantly predicted college students’ moral disengagement in high and low-level perspective taking, but the predictive effect of virtuous personality trait on moral disengagement was stronger for adolescents with high perspective taking (*b*_simple_ = −0.30, *p* < 0.001, 95% CI = [−0.39, −0.21]) than for adolescents with low perspective taking (*b*_simple_ = −0.13, *p* < 0.01, 95% CI = [−0.22, −0.03]). These results indicate that perspective taking moderates the indirect relationships between college students’ virtuous personality trait and online deviant behavior via moral disengagement, establishing the moderated mediation model. Therefore, Hypothesis 3 is supported.

### 3.5. Testing for the Moderated Moderated-Mediation Model

To analyze Hypothesis 4, this study tested the moderated moderated-mediation model by using Hayes’s PROCESS macro (Model 11) [53]. In this model, college students’ virtuous personality trait was the predictor, their level of perspective taking was the moderator, moral disengagement was the mediator, and online deviant behavior was the outcome variable. We predicted that college students’ gender would moderate the moderating effects of perspective taking on the association between virtuous personality trait and moral disengagement. Adolescent college students’ age and grade were included as covariates. The results showed a significant three-way interaction effect of college students’ virtuous personality trait, perspective taking, and gender on college students’ online deviant behavior via moral disengagement (*β* = 0.06, *p* < 0.05, 95% CI = [0.01, 0.12]; see Table 4). The results also showed that the index of moderated moderated-mediation model was significant (Index = 0.03, SE (Boot) = 0.01, 95% CI = [0.001, 0.05]). 

**Figure 3:**
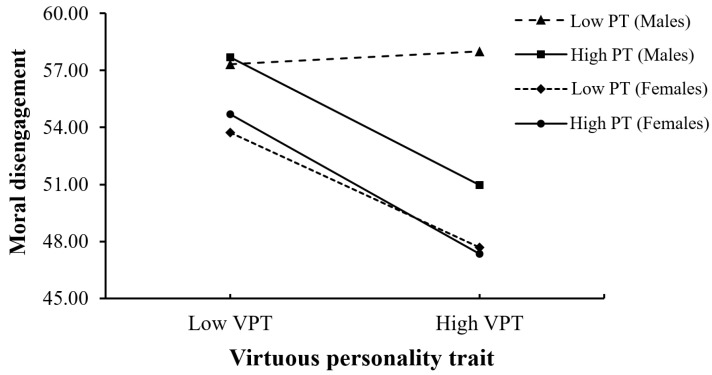
Moral disengagement as a function of VPT, PT, and gender.

According to the moderated moderated-mediation procedure [53], the present study first plotted the interaction effect (see Figure 3), and then examined the conditional effects of adolescent college students’ virtuous personality trait at values of the moderators (see Table 4) and the conditional indirect effects of college students’ virtuous personality trait on online deviant behavior (see Table 5). The results revealed that the effect of college students’ virtuous personality trait on online deviant behaviors via moral disengagement was more significant for men with high perspective taking than for those with low perspective taking (Index = −0.07, SE (Boot) = 0.02, 95% CI = [−0.12, −0.03]; see Table 5). Specifically, for men with high perspective taking, the results support the moderated moderated-mediation effect of moral disengagement (*β* = −0.27, *p* < 0.001, 95% CI = [−0.41, −0.13]; see Table 4). While, for men with low perspective taking, the results revealed that moral disengagement did not play a significant moderated moderated-mediating role (*β* = 0.09, *p* > 0.05, 95% CI = [−0.08, 0.27]; see Table 4). Furthermore, the effect of college students’ virtuous personality trait on online deviant behavior via moral disengagement was significant for women with a high level of perspective taking and for those with a low level of perspective taking. Specifically, college students’ virtuous personality trait had a stronger effect on online deviant behavior via moral disengagement for women with a high level of perspective taking (*β* = −0.30, *p* < 0.001, 95% CI = [−0.40, −0.19]; see Table 4) than those women with a low level of perspective taking (*β* = −0.20, *p* < 0.001, 95% CI = [−0.31, −0.10]; see Table 4), but the conditional effect difference was not significant (Index = −0.02, SE (Boot) = 0.01, 95% CI = [−0.04, 0.003]; see Table 5). Therefore, Hypothesis 4 is supported.

## 4. Discussion

The present study explored how and when the virtuous personality trait affects adolescent online deviant behavior by constructing a moderated moderated-mediation model. The results demonstrated that adolescent college students’ virtuous personality trait is negatively associated with their online deviant behavior. Furthermore, this study found that adolescents’ moral disengagement mediates the relationship between virtuous personality trait and online deviant behavior, and adolescents’ perspective taking plays a moderating role in the first stage of the indirect effect. Moreover, the present study also revealed that adolescent gender can moderate the moderating effect of perspective taking on the link between adolescents’ virtuous personality trait and moral disengagement. All of these findings will be discussed in the subsequent sections.

### 4.1. Relationship between Virtuous Personality Trait and Online Deviant Behavior

In recent years, adolescents’ online deviant behavior has attracted increasing attention from researchers [4,6,10,12,13]. How to reduce adolescents’ online deviant behaviors has become the focus of attention. Most of the previous studies focused on the risk factors (e.g., dark side of personality traits, peer alienation, and parental maltreatment) of adolescents’ online deviant behaviors [3,8,13]. However, according to the theory of positive youth development [18,19,20], we should not only pay attention to these risk factors, but also pay more attention to protective factors to reduce adolescents’ online deviant behaviors. Personality traits are relatively stable across time and context and can also play a role in the Internet environment [8]. In this study, we discovered that adolescent college students’ virtuous personality trait can be a protective factor, which can negatively predict online deviant behaviors significantly. Chinese Confucian culture has always attached great importance to the value of benevolence. As an embodiment of the value of benevolence, the virtuous personality trait is considered as a positive personality characteristic of Chinese culture [21]. Individuals with a high virtuous personality trait tend to have more positive behaviors and less problematic behaviors [21,22,23], which is still the case in the Internet environment [54]. Spontaneously, why virtuous personality trait can play a protective role in reducing adolescent college students’ online deviant behaviors is understandable. Therefore, the Hypothesis 1 is verified.

### 4.2. Mediating Role of Moral Disengagement

As far as we know, this research is the first to explore the association between adolescent college students’ virtuous personality trait and their online deviant behavior. Consistent with our hypothesis, we found that moral disengagement mediates the association between virtuous personality trait in college students and their online deviant behavior. That is, college students with higher virtuous personality trait are more likely to have lower levels of moral disengagement, which in turn leads to a decline in online deviant behavior. The results are consistent with many previous studies, which have identified that moral disengagement can serve as the mediating mechanism of some psychological variables affecting online deviant behaviors [25,34]. As a type of moral character, the virtuous personality trait can make individuals exhibit more cognitive processing tendencies and behavior patterns consistent with moral standards and norms [5,21,23]. Previous studies have discovered that kindness-related personality traits are significantly negatively correlated with moral disengagement [29,30]. Thus, individuals with high virtuous personality trait are less likely to exhibit moral disengagement strategies. Meanwhile, numerous of empirical studies have indicated that moral disengagement is an important antecedent variable of online deviant behaviors [26,55]. Consequently, moral disengagement can play a mediating role in the relationship between virtuous personality trait and online deviant behaviors. Therefore, Hypothesis 2 is verified.

### 4.3. Moderating Role of Perspective Taking and Gender

This research also discovered that college students’ perspective taking moderates the link between virtuous personality trait and moral disengagement, which is consistent with our hypothesis. We found that perspective taking can play an accelerating effect in the effects of college students’ virtuous personality trait on decreasing their online deviant behavior via moral disengagement. Compared with those college students with low levels of perspective taking, a high perspective taking level helps the college students enhance the weakened effects of virtuous personality trait on their moral disengagement. As a type of empathy, perspective taking has been confirmed to be negatively related to moral disengagement significantly [25,33]. That is, perspective taking can act as a protective factor [56]. Furthermore, this study found that virtuous personality trait can serve as a protective factor to reduce college students’ moral disengagement and online deviant behaviors. According to the protective-protective model [57], one protective factor can amplify the effect of other protective factors on an outcome variable. In this study, perspective taking and virtuous personality trait could help reduce college students’ moral disengagement in the network environment. For adolescents with both protective factors, perspective taking may strengthen the weakening effect of virtuous personality trait on college students’ moral disengagement and then reduce their online deviant behaviors. Therefore, the Hypothesis 3 is verified.

Moreover, previous studies have indicated that gender differences exist in many aspects of psychology and behavior [39]. For example, women usually tend to possess higher levels of perspective taking and virtuous personality trait than men [39,44]. As a consequence, the current study also constructed a moderated moderated-mediation model to test whether adolescent college students’ gender moderates the moderating effect of perspective taking on the link between virtuous personality trait and moral disengagement. The results revealed a significant three-way interaction among virtuous personality trait, perspective taking, and gender. Specifically, the moderating effect of perspective taking on the link between adolescent college students’ virtuous personality trait and moral disengagement was significant for men, but not for women. According to the evolutionary theory and Miller’s relational theory [39,43], women have higher kindness and a stronger desire to develop and maintain interpersonal relationships than men. Thus, women are less likely to exhibit moral disengagement, a tactic that can damage interpersonal relationships. Accordingly, the virtuous personality trait of women has a significant negative predictive effect on moral disengagement at high and low levels of perspective taking. However, men do not have a strong desire to establish and maintain interpersonal relationships and are more prone to moral disengagement [33,40,44]. Hence, only when men have a high level of perspective taking and can sincerely empathize with others will their virtuous personality trait significantly negatively predict their moral disengagement. Therefore, the Hypothesis 4 is verified.

### 4.4. Implications for Theory and Practice

There are several important theoretical and practical implications in this study. On the theoretical level, first of all, the current study is the first to explore and reveal the protective effect of adolescent college students’ virtuous personality trait on their online deviant behaviors. To some extent, the findings of this research validate the interpretation of online deviant behaviors by positive youth development theory, problem behavior theory and risk factors/protection factors model, and extend the application scope of these theories to the network world. Second, current study identified that moral disengagement has a mediating effect in the association between virtuous personality trait and adolescent college students’ online deviant behaviors, which can provide a new explanation path for the occurrence of adolescent college students’ online deviant behaviors. Last but not least, the study also revealed gender differences in the moderating effects of perspective taking. On the one hand, this result can help explain when adolescent college students’ virtuous personality trait has a stronger effect on online deviance behaviors; on the other hand, it also confirms and expands the evolutionary theory and Miller’s relationship theory.

On the practical level, the current study can provide several inspirations for the intervention and prevention of adolescent online deviant behaviors. First, the current study showed that virtuous personality trait is a significant protective factor for adolescent college students’ online deviant behaviors. Although personality traits are relatively stable, many personality theorists believe that individual personality traits can be further improved [58,59]. Previous studies have shown that kindness can be cultivated and promoted through education [60]. Hence, in the future, more attention should be paid to cultivating the kindness quality of adolescents to prevent online deviant behaviors. Second, this study demonstrated that moral disengagement is a mechanism that links adolescent college students’ virtuous personality trait to their online deviant behaviors. Given the hidden nature of the network environment, individuals are more likely to exhibit moral disengagement [7,25,33]. Thus, increasing deindividuation cues in the network may help adolescents decrease online deviant behaviors. Third, current study revealed that the moderating effects of adolescent college students’ perspective taking and gender are significant. The results of this study indicated that enhancing adolescents’ perspective-taking ability, especially the perspective-taking ability of men, could help reduce adolescents’ online deviant behaviors. 

### 4.5. Limitations and Future Directions

Although there are some interesting findings, the current study has several limitations that are worth noting. First, the present study is a cross-sectional design, which is essentially a correlation study, and no causal inference can be drawn. Future studies can adopt longitudinal design to confirm the causal relationship between variables. Second, this survey primarily uses college students as research samples to explore and uncover the influencing factors and psychological mechanism of adolescents’ online deviant behaviors. Future studies should consider expanding the sample by including primary and middle school students to disclose more comprehensively the characteristics of adolescents’ online deviant behaviors. Third, this study only collected data through the measurement method of subjective self-report of adolescents, which may affect the validity of the study. Future studies should consider a combination of self-evaluation, other evaluations, and other objective evaluation indicators to test the robustness of the results of this study. Finally, this study only explored the influence and mechanism of virtuous personality trait on adolescents’ online deviant behaviors from the individual level. Previous studies have indicated that some social environmental factors (e.g., family variables and peer variables) also have an impact on adolescents’ deviant behaviors [6,10,13,15]. Future studies could consider selecting variables from the social environment to explore further the influence mechanism of other variables on adolescents’ online deviant behaviors.

## 5. Conclusions

Virtuous personality trait is considered as a personality characteristic of Chinese culture, which can guide adolescents toward positive behaviors and reduce problem behaviors. Based on the theory of positive youth development, the present study first explored the relationship and psychological mechanism between virtuous personality trait and online deviant behavior of Chinese adolescent college students. The results of this study found that (1) virtuous personality trait was negatively correlated with online deviant behavior; (2) moral disengagement mediated the relationship between virtuous personality trait and online deviant behavior; (3) perspective taking moderated the first half stage of the mediation model in which college students’ virtuous personality trait influences online deviant behavior via moral disengagement; (4) college students’ gender moderated the moderating effect of perspective taking on the relationship between virtuous personality trait and moral disengagement. These findings can provide a positive and meaningful perspective for preventing and reducing adolescent problem behaviors and promoting adolescent healthy development.

## Figures and Tables

**Table 1:** Descriptive statistics and correlation matrix of all variables (N = 851).

Variables	*M*	*SD*	*Skewness*	*Kurtosis*	1	2	3	4	5
1. Gender	0.69	0.46	−0.80	−1.36	1.00				
2. Virtuous personality trait (VPT)	130.31	15.87	−0.12	0.16	0.12	1.00			
3. Moral disengagement (MD)	53.02	12.18	0.60	0.84	−0.18 ***	−0.24 ***	1.00		
4. Perspective taking (PT)	18.00	3.19	−0.25	0.10	−0.08 *	0.30 ***	−0.11 **	1.00	
5. Online deviant behavior (ODB)	57.85	16.33	1.40	2.44	−0.25 ***	−0.26 ***	0.45 ***	−0.05	1.00

*Note*. * *p* < 0.05; ** *p* < 0.01; *** *p* < 0.001. Men = 0, Women = 1.

**Table 2:** Testing the mediation effect of virtuous personality trait on online deviant behavior.

Variables	Model 1 (ODB)		Model 2 (MD)		Model 3 (ODB)	
	*β*	*t*	*β*	*t*	*β*	*t*
Age	0.03	0.76	−0.02	−0.40	0.04	1.02
Grade	0.03	0.67	0.02	0.45	0.02	0.53
VPT	−0.27	−7.99 ***	−0.23	−7.00 ***	−0.17	−5.43 ***
MD					0.41	13.24 ***
*R* ^2^	0.07		0.06		0.23	
*F*	21.67 ***		16.54 ***		63.40 ***	

*Note.* VPT = Virtuous personality trait; MD = Moral disengagement; ODB = Online deviant behavior; All variables in the model are standardized; *** *p* < 0.001.

**Table 3:** Testing the moderated mediation model.

Variables	Model 1 (MD)			Model 2 (AODB)		
	*β*	*t*	*95% CI*	*β*	*t*	*95% CI*
Age	−0.01	−0.35	[−0.09, 0.06]	0.04	1.02	[−0.03, 0.11]
Grade	0.01	0.34	[−0.06, 0.09]	0.02	0.53	[−0.05, 0.09]
VPT	−0.21	−6.09 ***	[−0.28, −0.14]	−0.17	−5.43 ***	[−0.23, −0.11]
PT	−0.05	−1.40	[−0.12, 0.02]	-	-	-
VPT × PT	−0.09	−2.93 **	[−0.14, −0.03]	-	-	-
MD	-	-	-	0.41	13.24 ***	[0.35, 0.47]
*R* ^2^	0.07			0.23		
*F*	12.12 ***			63.40 ***		
***Index of moderated-mediation***						
**Index**	**Boot SE**		**Boot LLCI**	**Boot ULCI**		
−0.04	0.01		−0.06	−0.02		

*Note.* VPT = Virtuous personality trait; MD = Moral disengagement; PT = Perspective taking; ODB = Online deviant behavior; All variables in the model are standardized; ** *p* < 0.01; *** *p* < 0.001.

**Table 4:** Testing the moderated moderated-mediation model.

Variables	Model 1 (MD)			Model 2 (AODB)		
	*β*	*t*	*95% CI*	*β*	*t*	*95% CI*
Age	−0.02	−0.51	[−0.10, 0.06]	0.04	1.02	[−0.03, 0.11]
Grade	0.01	0.38	[−0.06, 0.09]	0.02	0.53	[−0.05, 0.09]
VPT	−0.20	−5.80 ***	[−0.27, −0.13]	−0.17	−5.43 ***	[−0.23, −0.11]
PT	−0.06	−1.81	[−0.13, 0.01]	-	-	-
Gender	−0.21	−6.21 ***	[−0.28, −0.14]	-	-	-
VPT × PT	−0.09	−3.09 **	[−0.15, −0.03]	-	-	-
VPT × Gender	−0.08	−2.18 *	[−0.14, −0.01]	-	-	-
PT × Gender	0.07	2.20 *	[0.01, 0.14]	-	-	-
VPT × PT × Gender	0.06	2.17 *	[0.01, 0.12]	-	-	-
MD	-	-	-	0.41	13.24 ***	[0.35, 0.47]
*R* ^2^	0.11			0.23		
*F*	11.92 ***			63.40 ***		
**Conditional effects of the VPT at values of the moderators**						
**PT**	**Gender**	**Conditional effect**	**SE**	***t***	**LLCI**	**ULCI**
Low	Men	0.09	0.09	1.08	−0.08	0.27
Low	Women	−0.20	0.05	−3.74 ***	−0.31	−0.10
High	Men	−0.27	0.07	−3.66 ***	−0.41	−0.13
High	Women	−0.30	0.05	−5.56 ***	−0.40	−0.19

*Note.* VPT = Virtuous personality trait; MD = Moral disengagement; PT = Perspective taking; ODB = Online deviant behavior; all variables in the model are standardized; * *p* < 0.05; ** *p* < 0.01; *** *p* < 0.001.

**Table 5:** Conditional indirect effects of virtuous personality trait on online deviant behavior.

Perspective Taking	Gender	Effect	Boot SE	Boot LLCI	Boot ULCI
Low	Men	0.04	0.04	−0.04	0.11
Low	Women	−0.08	0.02	−0.13	−0.04
Medium	Men	−0.04	0.03	−0.09	0.02
Medium	Women	−0.10	0.02	−0.14	−0.07
High	Men	−0.11	0.03	−0.18	−0.05
High	Women	−0.12	0.02	−0.17	−0.08
**Indices of conditional moderated mediation by perspective taking**					
**Gender**		**Index**	**Boot SE**	**Boot LLCI**	**Boot ULCI**
Men		−0.07	0.02	−0.12	−0.03
Women		−0.02	0.01	−0.04	0.003

## Data Availability

The datasets generated during and/or analyzed during the current study are available from the corresponding author on reasonable request. The specific questionnaires (Chinese version) used in this study are available on request from the corresponding author.
